# A simple method for studying the molecular mechanisms of ultraviolet and violet reception in vertebrates

**DOI:** 10.1186/s12862-016-0637-9

**Published:** 2016-03-22

**Authors:** Shozo Yokoyama, Takashi Tada, Yang Liu, Davide Faggionato, Ahmet Altun

**Affiliations:** Department of Biology, Emory University, Atlanta, GA 30322 USA; Department of Physics, Fatih University, Istanbul, 34500 Turkey; Department of Genetics and Bioengineering, Fatih University, Istanbul, 34500 Turkey

**Keywords:** Visual pigments, UV and violet reception, Spectral tuning, Mutagenesis analyses, Hydrogen-bond network

## Abstract

**Background:**

Many vertebrate species use ultraviolet (UV) reception for such basic behaviors as foraging and mating, but many others switched to violet reception and improved their visual resolution. The respective phenotypes are regulated by the short wavelength-sensitive (SWS1) pigments that absorb light maximally (λ_max_) at ~360 and 395–440 nm. Because of strong epistatic interactions, the biological significance of the extensive mutagenesis results on the molecular basis of spectral tuning in SWS1 pigments and the mechanisms of their phenotypic adaptations remains uncertain.

**Results:**

The magnitudes of the λ_max_-shifts caused by mutations in a present-day SWS1 pigment and by the corresponding forward mutations in its ancestral pigment are often dramatically different. To resolve these mutagenesis results, the A/B ratio, in which A and B are the areas formed by amino acids at sites 90, 113 and 118 and by those at sites 86, 90 and 118 and 295, respectively, becomes indispensable. Then, all critical mutations that generated the λ_max_ of a SWS1 pigment can be identified by establishing that 1) the difference between the λ_max_ of the ancestral pigment with these mutations and that of the present-day pigment is small (3 ~ 5 nm, depending on the entire λ_max_-shift) and 2) the difference between the corresponding A/B ratios is < 0.002.

**Conclusion:**

Molecular adaptation has been studied mostly by using comparative sequence analyses. These statistical results provide biological hypotheses and need to be tested using experimental means. This is an opportune time to explore the currently available and new genetic systems and test these statistical hypotheses. Evaluating the λ_max_s and A/B ratios of mutagenized present-day and their ancestral pigments, we now have a method to identify all critical mutations that are responsible for phenotypic adaptation of SWS1 pigments. The result also explains spectral tuning of the same pigments, a central unanswered question in phototransduction.

**Electronic supplementary material:**

The online version of this article (doi:10.1186/s12862-016-0637-9) contains supplementary material, which is available to authorized users.

## Background

Early vertebrate ancestors used ultraviolet (UV) reception [1], which have been maintained in a wide range of modern species and play key roles in mate choice, foraging, predator avoidance, communication and migration [2–6]. In many other species, however, UV reception has been replaced by violet (or blue) reception during evolution [1]. Retinas of these animals do not receive UV light [7, 8] and are protected from UV damage and also their newly acquired violet reception have improved visual resolution and subtle contrast detection [9]. The avian ancestor also lost UV reception, but certain modern species regained it [1], which are again useful for various behaviors, including orientation based on the sun compass during migration [3, 6, 10].

UV and violet reception are regulated by the short wavelength-sensitive (SWS1) pigments that absorb light maximally (λ_max_) at ~360 and 395–440 nm, respectively [1]. To study the molecular basis of spectral tuning in a present-day pigment, vision scientists introduce mutations into orthologous as well as paralogous pigments and try to convert the λ_max_s of these mutants to the λ_max_ of interest [11–13]. However, identical mutations in different pigments can generate different λ_max_s and therefore variable sets of mutations can achieve similar λ_max_s [14, 15]. Similarly, to recapitulate the evolution of a certain present-day pigment, molecular evolutionists infer the evolution in a reverse fashion by introducing mutations into the present-day pigment, but the magnitudes of λ_max_-shift caused by these mutations and the corresponding forward mutations in its ancestral pigment can differ drastically [11–13, 16, 17]. Hence, using the traditional experimental approach, multiple sets of mutations can explain an observed λ_max_ and the evolutionary mechanisms inferred can be misleading or even erroneous [14, 15, 18, 19]. This “multiple-solution” problem occurs because 1) pigment-specific non-additive (epistatic) interactions are ignored and 2) the current mutagenesis experiments are used to search for “any” mutations that can achieve targeted λ_max_s. The first problem must be corrected by reconstructing appropriate ancestral pigments and manipulating them rather than present-day pigments [14, 15, 20, 21]. To address the second problem, we need to establish the one-to-one relationship between the SWS1 pigment and the dichotomous phenotypes; for that, a new approach is required.

Each visual pigment consists of an opsin and the retinal, either 11-*cis*-retinal or 11-*cis*-3, 4-dehydroretinal [22, 23]. At the chemical level, each visual pigment consists of a mixture of pigments with protonated Schiff base (SB) nitrogen-linked retinals (PSBR) and those with unprotonated SB nitrogen-linked retinals (SBR). When the SBR form is energetically more stable than PSBR, the SWS1 pigment is UV-sensitive; otherwise it is violet-sensitive [24–26]. The protonation status of SB nitrogen is influenced strongly by the hydrogen-bond network (HBN) that is formed by amino acids at sites 86, 90, 113, 114, 118 and 295 and two water molecules [25]; throughout the paper, the amino acid site numbers follow those of bovine rhodopsin (GenBank accession no. M21606). Therefore, the HBN structure offers an opportunity to establish the one-to-one relationship between a SWS1 pigment and its dichotomous phenotype and identify all critical mutations unambiguously.

## Results

### Ancestral pigments

**Fig. 1:**
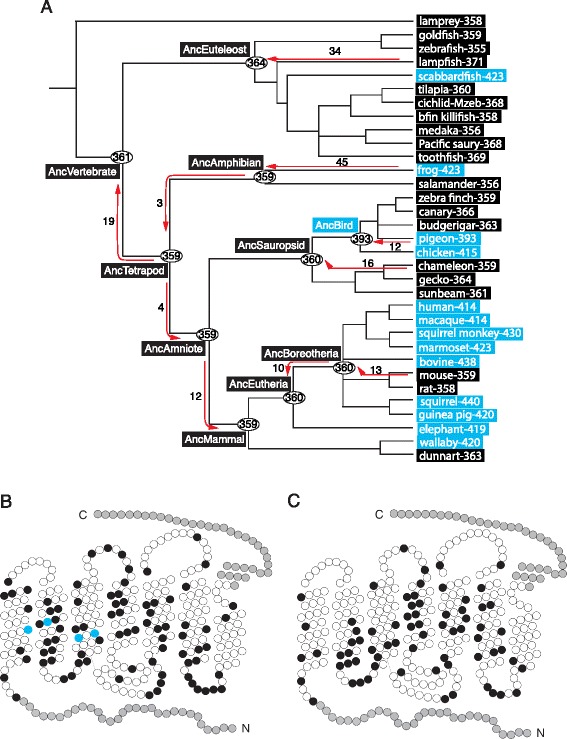
Construction of ancestral SWS1 pigments from 33 present-day sequences. (**a**) A composite phylogenetic tree with the λ_max_s at 10 critical nodes. The numbers beside various branches indicate the numbers of mutations introduced into present-day and ancestral pigments. Arrows indicate the direction of the reconstruction of ancestral pigments. The numbers after each organism (and their SWS1 pigments) indicate their λ_max_s. UV- and violet-sensitive pigments are distinguished by black and blue rectangles, respectively. (**b**) The neutral and adaptive mutations in ancestral pigments (black and blue circles, respectively). (**c**) Statistically inferred ancestral amino acids that have PP < 95 % (in black circles). The 2-D models are after Palczewski [97]

Previously, we inferred the amino acid sequences of ancestral pigments by applying maximum likelihood-based Bayesian method (PAML [27]) to a phylogenetic tree of 21 SWS1 pigments and genetically engineered pigments of the ancestors of 1) jawed vertebrates (AncVertebrate), 2) Tetrapods (AncTetrapod), 3) Amphibians (AncAmphibian), 4) Amniotes (AncAmniote), 5) Sauropsids (AncSauropsid), 6) Aves (AncBird) and 7) Boreoeutheria (AncBoreotheria) [1]. Here we applied the PAML with JTT and WAG substitution models to a new phylogenetic tree of 33 representative SWS1 pigments (Fig. 1a) and inferred all ancestral pigments. The amino acid sequences inferred are highly consistent and have very similar posterior probabilities (PP). For AncVertebrate, for example, the two models predict the same amino acids at 244 (86 %) out of a total of 282 sites considered (see below) with PP ≥ 0.95 and at 23 of the remaining 38 sites with PP ≥ 0.70; different amino acids are predicted only at three sites (Additional file 1: Table S1).

Since JTT and WAG models predict very similar ancestral sequences, we used the amino acid sequences predicted by JTT model (Additional file 2: Figure S1) and introduced the necessary amino acid changes into the internal segment (sites 31–312) in the pMT5 containing the N- and C-termini of the chameleon-359 (for the justification, see Methods) and engineered AncVertebrate, AncTetrapod, AncAmphibian, AncAmniote as well as those of Euteleosts (AncEuteleost), Mammals (AncMammal) and Eutherians (AncEutheria) (Additional file 2: Figure S1; Fig. 1a). The in vitro assays [28] show that the λ_max_s of AncVertebrate, AncTetrapod, AncAmphibian and AncAmniote which are based on the two different phylogenetic trees have λ_max_s of ~ 360 nm and, furthermore, with the exception of AncBird-393, the ancestral pigments (AncVertebrate-361, AncEuteleost-364, AncTetrapod-359, AncAmphibian-359, AncAmniote-359, AncSauropsid-360, AncMammal-359, AncEutheria-360 and AncBoreotheria-360 [29]) are all UV-sensitive, where the numbers after pigment names indicate their λ_max_s (Fig. 1a, Additional file 3: Figure S2).

When the amino acids at a total of 282 sites of the 10 ancestral SWS1 pigment sequences are compared, we can find 85 polymorphic sites (Fig. 1b, Additional file 2: Figure S1), among which only amino acid changes F49V, F86S, L116V and S118A (or F49V/F86S/L116V/118S) cause the significant λ_max_-shift between AncSauropsid-360 and AncBird-393 [1]. Hence, amino acid changes at 81 out of the 85 sites (~95 %) do not cause any significant λ_max_-shift and may be considered as “selectively neutral” changes (Fig. 1b). Virtually identical proportions of “neutral” changes have been observed for paralogous RH1 (or rhodopsin) and middle and long wavelength-sensitive (M/LWS) pigments [30, 31]. It is also comforting to learn that, despite having a total of 70 amino acid sites with PP < 0.95, none of the λ_max_s of the 10 ancestral pigments are affected significantly by the uncertainty involved in the statistical inference (Fig. 1c).

### Mutagenesis-induced phenotypes; a small number of mutations

**Fig. 2:**
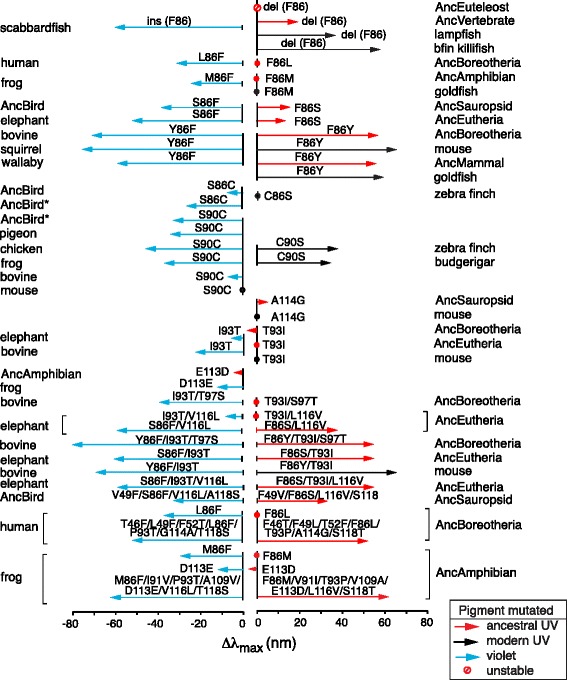
The λ_max_-shifts generated by various mutations. The lengths of arrows represent the λ_max_-shifts and filled circles indicate that no λ_max_-shift occurred. Red and black arrows show the mutational effects of ancestral and present-day UV pigments, respectively, whereas blue arrows indicate those of violet pigments. The different λ_max_-shifts of bovine mutants with Y86F have been evaluated by using dark spectra [84] and dark–light spectra [92] and only the former result is shown. AncBird*-393 is identical to AncSauropsid-360 with mutations F49V/F86S/L116V/S118A

To better understand the nature of the currently available mutagenesis results of SWS1 pigments [13–15], we added our own 23 new data (Methods). Using these old and new mutagenesis results, we first establish that the λ_max_-shifts caused by a small number of mutations often deviate drastically from the additive mutational effects and depend strongly on their molecular backgrounds (Fig. 2, Additional file 4: Table S2). Probably the most unusual mutation is the deletion of the codon 86, which encoded F86 in AncVertebrate-361, in scabbardfish-423 of *Lepidopus fitchi*. When we consider present-day pigments, the violet-sensitivity of scabbardfish-423 and the UV-sensitivities of bfin killifish-355 (*Lucania goodie*) and lampfish-371 (*Stenobrachius leucepsarus*) can be interchanged largely by deleting F86 from the UV pigments and inserting F86 into scabbardfish-423, respectively, which suggest that F86 deletion has significantly contributed to scabbardfish-423 evolution. However, the λ_max_-shifts caused by the two deletion mutants differ by ~20 nm and, furthermore, when F86 is deleted from AncVertebrate-361, the λ_max_ of the mutant is 43 nm shorter than that of scabbardfish-423 [26]. Much to our surprise, when F86 is deleted from AncEuteleost-364, which is more closely related to scabbardfish-423 than AncVertebrate-361 (Fig. 1a), the mutant pigment becomes structurally unstable and its λ_max_ cannot be evaluated. All of these results show that additional mutations are involved in the scabbardfish-423 evolution.

One major characteristic of the mutagenesis results is that the magnitude of a λ_max_-shift (or Δλ_max_) caused by mutations tends to be much smaller in UV pigments than in violet pigments. We can see this in the 11 sets of comparisons (Fig. 2): 1) F86 deletion in AncVertebrate-361 and F86 insertion in scabbardfish-423 (Δλ_max_ = 19 vs −60 nm, respectively); 2) F86L in AncBoreotheria-360 and L86F in human-414 (0 vs −32 nm); 3) F86M in AncAmphibian-359 and M86F in frog-423 (*Xenopus laevis*) (2 vs −25 nm); 4) F86S in AncSauropsid-360 and AncEutheria-360 and S86F in AncBird-393 and elephant-419 (*Loxodonta africana*) (17 vs −38 and 14 vs −52 nm, respectively); 5) F86Y in AncBoreotheria-360 and Y86F in bovine-438 (*Bos taurus*) and squirrel-440 (*Sciurus carolinensis*) (48 vs −71 ~ −76 nm); 6) C86S in zebra finch-359 (*Taeniopygia guttata*) and S86C in the functionally equivalent violet pigments (AncBird-393 and AncSauropsid-360 with F49V/F86S/L116V/118S, or AncBird*-393) (1 vs −7 or −27 nm); 7) T93I in AncBoreotheria-360 and I93T in bovine-438 (−4 vs −22 nm); 8) T93I/S97T in AncBoreotheria-360 and I93T/T97S in bovine-438 (1 vs −39 nm); 9) T93I/L116V in AncEutheria-360 and I93T/V116L in elephant-419 (0 vs −8 nm); 10) F86S/L116V in AncEutheria-360 and S86F/V116L in elephant-419 (38 vs −59 nm) and 11) F86Y/T93I/S97T in AncBoreotheria-360 and the reverse changes in bovine-438 (48 vs −79 nm).

Hence, epistatic interactions tend to operate more strongly in UV pigments than in violet pigments. Furthermore, identical mutations cause variable λ_max_-shifts among orthologous pigments. S90C contributed significantly to the evolution of UV-sensitivities of some modern avian pigments (e.g. zebra finch-359, canary-366 (*Serinus canaria*) and budgerigar-360 (*Melopsittacus undulatus*)) from the violet-sensitive AncBird-393 [1, 32, 33] (Fig. 1a). S90C in AncBird*-393, pigeon-393, chicken-415, frog-423 and bovine-438 decreases their λ_max_s by 7–46 nm, whereas the identical mutation causes no λ_max_-shift in mouse-359 (Additional file 4: Table S2). Variable Δλ_max_s with the same mutations can also be found in 1) deletion of F86 from AncVertebrate-361, lampfish-371 and bfin killifish-354 (Δλ_max_ = 19–58 nm), 2) S86F in AncBird-393 and elephant-419 (Δλ_max_ = −38 and −52 nm, respectively), 3) F86Y in AncBoreotheria-360, mouse-359 and goldfish-360 (Δλ_max_ = 48–66 nm), 4) S86C in phenotypically identical AncBird-393 and AncBird^*^-393 (Δλ_max_ = −7 and −27 nm, respectively) and 5) I93T in AncEutheria-360, AncBoreotheria-360, mouse-359, elephant-419 and bovine-438 (∣Δλ_max_∣ = 0–22 nm).

In summary, 1) identical mutations can cause, sometimes drastically, different λ_max_-shifts, 2) forward and reverse mutations can shift the λ_max_ by different magnitudes to the opposite directions, or even in the same direction, and 3) UV pigments, particularly ancestral UV pigments, tend to be less responsive to mutations than violet pigments to the corresponding reverse changes. Two sets of forward and reverse mutations shift the λ_max_ in the same direction: 1) T93I in AncBoreotheria-360 and I93T in elephant-419 and bovine-438 and 2) E113D in AncAmphibian-359 and D113E in frog-423 (Additional file 4: Table S2). The differential effects of forward and reverse mutations clearly show that the evolutionary mechanisms of UV and violet reception must be studied by using ancestral pigments rather than present-day pigments. One notable exception is Y86F in wallaby-420 (*Macropus eugenii*) and F86Y in AncMammal-359, which fully interchange the two original λ_max_s (Fig. 2; Additional file 4: Table S2).

At the chemical level, each SWS1 pigment consists of a mixture of PSBR and SBR (see Background). The major λ_max_-shifts of SWS1 pigments are caused by changes in the relative ground-state energies of the pigments with the two retinal groups. The calculated relative ground-state energies of a SWS1 pigment with SBR subtracted from that with PSBR (ΔE) is positive (varying between 2.5 and 8.3 kcal/mol) for a UV pigment while it is negative for a violet pigment (varying between −2.4 and −5.6 kcal/mol) [25]. The wider ΔE range explains the functionally conservative nature of UV pigments.

### Multiple mutations

As the number of critical mutations identified increases, the magnitudes of λ_max_-shifts caused by forward and reverse mutations tend to become similar. Since epistatic interactions are reflected better by multiple mutations than by single mutations, this observation may be expected. This trend can be seen in 1) F86S/T93I in AncEutheria-360 and S86F/I93T in elephant-419 (Δλ_max_ = 55 vs −60 nm, respectively), 2) F86Y/T93I in mouse-359 and Y86F/I93T in bovine-438 (66 vs −68 nm) and 3) F86S/T93I/L116V in AncEutheria-360 and the reverse mutations in elephant-419 (50 vs −59 nm) (Fig. 2, Additional file 4: Table S2). We can find three examples of excellent symmetry between the λ_max_-shifts caused by forward mutations in an ancestral pigment and reverse mutations in a corresponding present-day pigment: 1) F49V/F86S/L116V/S118A in AncSauropsid-360 and the reverse mutations in AncBird-393 (33 vs −33 nm); 2) F86M/V91I/T93P/V109A/E113D/L116V/S118T in AncAmphibian-359 and the reverse mutations in frog-423 (62 vs −68 nm) and 3) F46T/F49L/T52F/F86L/T93P/A114G/S118T in AncBoreotheria-360 and the reverse mutations in human-414 (52 vs −54 nm) (Fig. 2).

The goal of all of these mutagenesis analyses is to find the molecular mechanisms of spectral tuning and evolution of a present-day pigment. A weakness of this traditional approach becomes apparent from the mutagenesis analyses of elephant-419 evolution. F86S/T93I in AncEutheria-360 and S86F/I93T in elephant-419 achieve Δλ_max_s of 55 and −60 nm, respectively (Additional file 4: Table S2), which interchange the λ_max_s of the two pigments reasonably well and elephant-419 seems to have evolved from AncEutheria-360 by F86S/T93I. However, elephant-419 has incorporated 14 additional mutations and AncEutheria-360 with F86S/T93I/L116V attains a Δλ_max_ of 50 nm (Additional file 4: Table S2), which moves further away from the λ_max_ of elephant-419, which show that neither F86S/T93I nor F86S/T93I/L116V explain elephant-419 evolution. Hence, to identify all critical mutations, it is necessary, but not sufficient, to manipulate and compare the λ_max_s of present-day pigments and their ancestral pigments. To alleviate this type of problem, we may check whether mutations that attained the desired λ_max_-shift also achieve the key protein structural change.

### Molecular modelling of Hydrogen-Bond Network (HBN): AMBER models

**Fig. 3:**
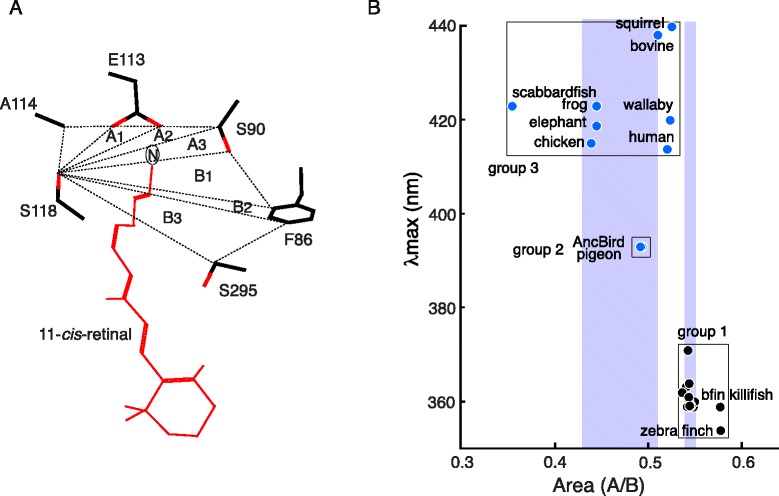
The HBN regions of SWS1 pigments. (**a**) Area A (A_1_-A_3_) contains SB nitrogen and the counter-ion E113 and area B (B1-B3) includes two water molecules. The example shows the six amino acids of AncVertebrate-361 and the 11-*cis*-retinal with SB nitrogen (N). Dotted lines indicate the shortest distances between two amino acids. (**b**) The λ_max_s and A/B ratios of the ancestral and present-day pigments. UV pigments are shown by black circles (group 1), whereas the violet-sensitive AncBird-393 and pigeon-393 (group 2) and the others (group 3) are shown by blue circles. The widths of light blue rectangles show the 95 % confidence intervals of the A/B ratios for group 1 (0.54–0.55) and group 3 (0.43–0.51), whereas the corresponding intervals for group 2 is a narrow strip at around 0.49 (not shown) (Additional file 5: Table S3). Scabbardfish-423 lacks F86 and the most closely located amino acid to the HBN region is V91 and therefore its A/B ratio is evaluated by considering V91 in place of F86

We divided the HBN region into two parts: one area formed by amino acids at sites 90, 113 and 118 (area A) and another area determined by those at sites 86, 90 and 118 and 295 (area B), each of which is further subdivided into three parts A_1_-A_3_ and B_1_-B_3_ (Fig. 3a). Since the amino acids at sites 113 and 114 are bonded to each other, their effects on the HBN structure and λ_max_-shift are correlated [25]. Therefore, one of these sites, i.e., 113, is included in area A. The distance between SB nitrogen that lies in the area A (Fig 3a) and the counterion E113 in AncVertebrate-361 is 1.99 Å, but the corresponding distances in AncBird-393 and human-414 are ~1.80 Å [25]. The wider distances contribute in making the SBN forms of UV pigments energetically more stable than PSBR forms, or vice versa for violet pigments. As the distance between E113 and the SB nitrogen decreases, the A/B ratio tends to decrease and the λ_max_ of a pigment is expected to increase (Fig. 3a).

For five representative pigments (AncVertebrate-359, AncBird-393, zebra finch-359, budgerigar-363 and human-414), initial structures were obtained from their amino acid sequences through SWISS MODEL homology modelling [34] using the structure of bovine rhodopsin (pdb code: 1U19) as a template. After adding hydrogen atoms and fully optimizing the modelled structures at pure AMBER96 force field level, we evaluated the sizes of areas A and B as well as A/B ratios.

The results show that UV pigments (AncVertebrate-359, zebra finch-359 and budgerigar-363) have A/B ratios of 0.507–0.527, followed by 0.421 of AncBird-393 and 0.406 of human-414, in that order, which show an inverse relationship between the A/B ratio and λ_max_ of a visual pigment (Table 1). To relate such structural variations with the λ_max_ values of the visual pigments, we performed multivariable regression analysis between the λ_max_ values (dependent variable) and structural parameters like A/B and B (independent variables) and obtained a relationship, λ_max_ = 390.33–357.284 x (A/B) + 2.345 x B. The λ_max_s predicted from this formula are very close to the actual values determined by the *in-vitro* assay (*R*^2^ = 0.969) and standard error (*S* = 6.065) and the mean absolute error (*MAE* = 3.085) are satisfactorily small (Table 1). Hence, the λ_max_ of a pigment decreases as the A/B ratio increases and B value decreases.

**Table 1:** Comparison of A/B values of HBNs using SWISS and AMBER models

					λ_max_ (nm)	
Pigment	Model	A (Å^2^)	B (Å^2^)	A/B	Observed	Predicted
AncVertebrate-361	AMBER	36.033	68.313	0.527	361	362
	SWISS	32.057	59.109	0.542		
Zebra finch-359	AMBER	34.166	65.783	0.519	359	359
	SWISS	36.937	64.031	0.577		
Budgerigar-363	AMBER	32.684	64.454	0.507	363	360
	SWISS	35.072	65.602	0.535		
AncBird-393	AMBER	28.598	67.945	0.421	393	399
	SWISS	33.396	67.758	0.493		
Human-414	AMBER	28.332	69.781	0.406	414	409
	SWISS	31.422	60.574	0.519		

### Simpler HBN models: SWISS models

The A/B ratios were also obtained directly from SWISS MODEL method considering only the amino acid sequences. We refer to the HBN structures before and after AMBER geometry optimizations as SWISS models and AMBER models, respectively (Table 1). The A/B ratios of SWISS models are larger than those of AMBER models and the overestimation is largest for human-414. Because of this, the inverse relationship between A/B ratio and λ_max_ does not hold for the less accurate SWISS models of the five pigments. However, when we consider the SWISS models of the 10 ancestral and all currently known 33 present-day SWS1 pigments, they can be distinguished roughly into three groups: 1) the A/B ratios of the SWISS models of the UV pigments with λ_max_s of 354–371 nm (0.547 ± 0.003, group 1) are larger than those of AncBird-393 and pigeon-393 (0.492 ± 0.0003, group 2), which tend to be larger than the A/B ratios of violet pigments (0.469 ± 0.021, group 3) (Fig. 3b, Additional file 5: Table S3). Like those of AMBER models, the smallest A/B ratios of the group 3 (or violet) pigments are caused by the compressed A region plus the expanded B region and the intermediate A/B ratios of the SWISS models of group 2 pigments come from an expanded B region (Additional file 5: Table S3).

Human-414, Squirrel-440, bovine-438 and wallaby-420 have much larger A/B ratios than the rest of the group 3 pigments; similarly, zebra finch-359 and bfin killifish-355 have much larger A/B ratios than the other group 1 pigments (Fig. 3b, Additional file 5: Table S3). During the evolution of human-414 from AncBoreotheria-360, three critical changes (F86L, A114G and S118T) have been incorporated in the HBN region. These changes make the compression of A region and expansion of B region in human-414 less effective in the SWISS models than in AMBER models and generate the higher A/B ratio of its SWISS model (Table 1). For the same reason, F86Y in squirrel-440, bovine-438 and wallaby-420 as well as F86C and S90C in zebra finch-359 and S118A in bfin killifish-355 have generated the large A/B ratios of their SWISS models. The smallest A/B ratio of scabbardfish-423 comes from its unique protein structure, in which V91 needs to be considered in place of F86.

The major advantage of using the less accurate SWISS models is that they are readily accessible to everyone and, importantly, the A/B ratios of the SWISS models of UV pigments (0.535–0.577) can still be distinguished from those of violet pigments (0.355–0.526) (Fig. 3b). In analysing SWS1 pigments, the variable λ_max_s and A/B values within each of the three pigment groups are irrelevant because we are concerned mainly with the major λ_max_-shifts among UV pigments (group 1), AncBird-393 (group 2) and violet pigments (group 3): group 1 → group 2, group 1 → group 3, group 2 → group 1 and group 2 → group 3 (Fig. 1a). For each of these phenotypic adaptive processes [1], we can establish the one-to-one relationship between A/B ratios and dichotomous phenotypes of SWS1 pigments.

### Criteria for acceptable mutagenesis results

To examine whether or not the mutagenesis result of a certain present-day pigment reflects the epistatic interactions correctly, we evaluate the λ_max_ and A/B ratio of its ancestral pigment subtracted from those of a mutant pigment (denoted as d(λ_max_) and d(A/B), respectively). Similarly, the validity of the mutagenesis result of an ancestral pigment can be examined by evaluating its d(λ_max_) and d(A/B) values by considering the λ_max_ and A/B ratio of the corresponding present-day pigments. Following the traditional interpretation of mutagenesis results, it seems reasonable to consider that present-day and ancestral mutant pigments fully explain the λ_max_s of the target (ancestral and present-day) pigments when ∣d(λ_max_)∣ ≤ 3 ~ 5 nm, depending on the magnitudes of total λ_max_-shift considered. Following the mutagenesis results of wallaby-420, AncBird-393, frog-423 and human-414 (see below), the A/B ratio of the target pigment may be considered to be fully converted when ∣d(A/B)∣ ≤ 0.002.

### Searching for the critical mutations in SWS1 pigments

Considering d(λ_max_) and d(A/B) together, mutagenesis results of SWS1 pigments can be distinguished into three classes: 1) amino acid changes satisfy ∣d(λ_max_)∣ ≤ 3 ~ 5 nm and ∣d(A/B)∣ ≤ 0.002 (class I); 2) those satisfy only ∣d(λ_max_)∣ ≤ 3 ~ 5 nm (class II) and 3) those satisfy neither conditions (class III) (Table 2, Additional file 5: Table S3). When only a small number of mutations are considered, class I includes F86 insertion in scabbardfish-423 and Y86F in wallaby-420, both achieving d(λ_max_) = 2 nm and d(A/B) = 0.001. However, the F86 deletion mutants of AncVertebrate-361, lampfish-371 and bfin killifish-355 all belong to class III, confirming that scabbard-423 did not evolve by F86 deletion alone. On the other hand, F86Y in AncMammal-359 belongs to class I, establishing that wallaby-420 indeed evolved from AncMammal-359 by F86Y alone. Compared with these two examples, Y86F in squirrel-440 and F86Y in AncBoreotheria-360 belong to classes II and III, respectively, showing that squirrel-440 evolution did not occur by F86Y alone.

**Table 2:** Comparisons of d(λ_max_) and d(A/B) for different sets of pigments

Pigment	Mutation	d(λ_max_) (nm)	d(A/B)	Class
AncVertebrate-361	F86 deletion	−43	−0.004	III
AncEuteleost-364	F86 deletion	ND		
Lampfish-371	F86 deletion	−15	0.003	III
bfin killifish-355	F86 deletion	6	0.028	III
scabbardfish-423	F86 deletion	2	0.001	I
AncBoreotheria-360	F86L	−54	−0.034	III
Human-414	L86F	22	−0.005	III
AncAmphibian-359	F86M	−62	0.036	III
Frog-423	M86F	39	−0.042	III
AncBoreotheria-360	F86Y	−30	0.020	III
Bovine-438	Y86F	7	−0.018	III
AncBoreotheria-360	F86Y	−32	0004	III
Squirrel-440	Y86F	4	−0.004	II
AncMammal-359	F86Y	−4	−0.001	I
Wallaby-420	Y86F	2	0.001	I
AncEutheria-360	F86S	−45	0.018	III
	F86S/T93I	−4	0.015	II
	F86S/T93I/L116V	−11	0.005	III
Elephant-419	S86F	7	−0.009	III
	S86F/I93T	−1	−0.019	II
	S86F/I93T/V116L	0	0.005	II
AncSauropsid-360	F49V/F86S/L116V/S118A	0	−0.001	I
AncBird-393	V49F/S86F/V116L/A118S	0	0.001	I
AncAmphibian-359	F86M/V91I/T93P/V109A/E113D/L116V/S118T	−2	0.002	I
Frog-423	M86F/I91V/P93T/A109V/D113E/V116L/T118T	−4	0.001	I
AncBoreotheria-360	F46T/F49L/T52F/F86L/T93P/A114G/S118T	−2	0.0	1
Human-414	T46F/L49F/F52T/L86F/P93T/G114A/T118S	0	−0.001	1

Class I also includes three sets of reverse mutations: 1) V49F/S86F/V116L/A118S in AncBird-393, 2) M86F/I91V/P93T/A109V/D113E/V116L/T118S in frog-423 and 3) T46F/L49F/F52T/L86F/P93T/G114A/T118S in human-414. The corresponding forward mutations in AncSauropsid-360, AncAmphibian-359 and AncBoreotheria-360 also belong to class I (Table 2). Hence, AncBird-393 evolved from AncSauropsid-360 by four mutations, while frog-423 and human-414 evolved from their ancestral pigments by a different set of seven mutations. On the other hand, despite their significant magnitudes of λ_max_-shifts, individual mutations L86F in human-414 (Δλ_max_ = −32 nm and d(λ_max_) = 22 nm) and M86F in frog-423 (Δλ_max_ = −25 nm and d(λ_max_) = 39 nm) belong to class III (Additional file 4: Table S2). Moreover, Y86F in bovine-438 decreases the λ_max_ by ~70 nm, but this mutation (d(λ_max_) = 7 nm) still belongs to class III and furthermore class III status of F86Y in AncBoreotheria-360 shows that the evolutionary mechanism of bovine-438 is still unsolved (Table 2).

Among the three classes, class II is particularly disconcerting because even when the λ_max_s of present-day pigments can be converted to those of their ancestral pigments, these mutations do not achieve the key protein structural changes. Class II includes Y86F of squirrel-440 as well as S86F/I93T and S86F/I93T/V116L of elephant-419 (Table 2). Hence, either additional mutations can be involved or they might not have played significant roles during evolution (see Discussion). As suspected, class III includes many single mutations, which are represented by such mutations as L86F in human-414, M86F in frog-423, Y86F in bovine-438 and S86F in elephant-419.

In summary, the goal of studying molecular basis of spectral tuning in a present-day pigment is to identify mutations that generated its λ_max_, while the mechanism of phenotypic adaptation of the same pigment is to find specific mutations that generated the λ_max_ during evolution. These questions address the same phenomenon and can be solved simultaneously; for the latter problem, however, it would also be necessary to establish the relationship between the phenotypic changes and the changes in the organisms’ new environments (see the next section). Hence, among all mechanisms of spectral tuning and adaptive evolution of SWS1 pigments proposed to date, only those for AncBird-393, frog-423, human-414 and wallaby-420 can be supported.

## Discussion

Mutations in different molecular backgrounds can differ significantly in their contribution to phenotypic adaptation [14, 15]. Here we have seen that 1) mutagenesis results of present-day SWS1 pigments are highly pigment-specific and 2) the one-to-one relationship holds between A/B ratios of HBN region and dichotomous phenotypes (UV- and violet-sensitivities) of SWS1 pigments. We then developed a method for identifying all critical mutations that generated the λ_max_s of present-day pigments by interchanging the λ_max_s and A/B ratios of the present-day and their ancestral pigments. In applying this approach, the following three features are critical to keep in mind.

First, it is imperative to reconstruct proper ancestral pigments and manipulate them. To see the necessity of manipulating ancestral molecules and their phenotypes, we revisit the evolution of elephant-419 from AncEutheria-360. At present, this process is explained best by F86S/T93I/L116V, which are responsible for about 85 % of the entire λ_max_-shift and the triple mutant in AncEutheria-360 achieve neither the λ_max_ nor A/B ratio of elephant-419 (class III), but the reverse mutant of elelphant-419 attains the λ_max_ of AncEutheria-360, but not the A/B ratio (class II mutations) (Table 2). Introducing all possible combinations of the three mutations into elephant-419 and applying a linear model to their λ_max_s and that of elephant-419 (λ_elephant_), the individual and epistatic effects of these mutations on the λ_max_-shift (θ) were evaluated (Table 3). The results show that the major contributor is S86F (θ_S86F_ = −52 nm) and the effects of forward mutations in AncEutheria-360 may be inferred by reversing the sign of this θ value. Alternatively, S86F, I93T, V116L, S86F/I93T, S86F/V116L, T93I/V116L and S86F/I93T/V116L in elephant-419 may be regarded as functionally equivalent to T93I/L116V, F86S/L116V, F86S/T93I, L116V, T93I, F86S in AncEutheria-360 and the ancestral pigment, respectively. Then again, θ_F86S_ (51 nm) has a major impact in the elephant-419 evolution.

**Table 3:** Effects of mutations on the λ_max_-shift

Pigment	Mutation	λ_max_ (nm)	λ_max_ and θ (nm)	
Elephant-419	–	419	λ_elephant_ = 419	λ_AncEutheria_ = 360
	S86F	367	θ_S86F_ = −52	θ_F86S_ = 51
	Ι93Τ	413	θ_I93T_ = −6	θ_T93I_ = 0
	V116L	416	θ_V116L_ = −3	θ_L116V_ = −1
	S86F/I93T	359	θ_S86F/I93T_ = −2	θ_F86S/T93I_ = 5
	S86F/V116L	360	θ_S86F/V116L_ = −4	θ_F86S/L116V_ = 3
	T93I/V116L	411	θ_I93T/V116L_ = 1	θ_T93I/L116V_ = 8
	S86F/I93T/V116L	360	θ _S86F/I93T/V116L_ = 7	θ _F86S/T93I/L116V_ = −7
AncEutheria-360	−	360	λ _AncEutheria_ = 360	
	F86S	374	θ_F86S_ = 14	
	T93I	360	θ_T93I_ = 0	
	L116V	359	θ_L116V_ = −1	
	F86S/T93I	415	θ_F86S/T93I_ = 41	
	F86S/L116V	398	θ_F86S/L116V_ = 25	
	T93I/L116V	360	θ_T93I/L116V_ = 1	
	F86S/T93I/L116V	410	θ_F86S/T93I/L116V_ = −30	

Consequently, elephant-419 seems to have evolved mostly by F86S. However, this conclusion is incorrect. That is, when we introduce the corresponding forward mutations into AncEutheria-360, epistatic interactions (θ_F86SxT93I_ = 41 nm, θ_F86SxL113V_ = 25 nm and θ_F86SxT93IxL113V_ = −30 nm) have major impacts and the F86S-effect (14 nm) becomes less significant (Table 3), again showing that epistatic interactions are much stronger in the UV pigment than in elephant-419. This example demonstrates that the spectral tuning and evolutionary mechanism of a present-day pigment must be studied by manipulating its ancestral pigment.

Second, the A/B ratio can be useful for checking whether certain mutations that cause significant λ_max_-shifts were actually used for phenotypic (or functional) changes. For example, S86C and S90C in AncBird*-393 decrease the λ_max_ to 366 and 360, respectively. In theory, both mutations explain the reversion from violet reception to UV reception in certain modern avian species. When the respective mutants are compared to budgerigar-359, d(A/B) values are 0.485–0.535 (= −0.050) and 0.528–0.535 (= −0.007); similarly, when they are compared to zebra finch-359, the d(A/B) values are 0.485–0.577 (= −0.092) and 0.528–0.577 (= −0.049), respectively (Additional file 4: Table S2 and Additional file 5: Table S3). In reality, therefore, the S86C and S90C mutants belong to classes III and II, respectively; moreover, the smaller ∣d(λ_max_)∣ and ∣d(A/B)∣ values suggest that S90C, not S86C, has contributed to the actual evolution of the avian UV pigments. Indeed, phylogenetic analyses strongly suggest that S90C preceded S86C and the effect of the latter mutation seems to have been insignificant during evolution [1].

Third, as suggested by S86C and S90C, d(λ_max_) and d(A/B) of a pigment are affected strongly by the order of mutation accumulations. For example, the seven critical mutations in AncAmphibian-359 and those in AncBoreotheria-360 shift the λ_max_ individually only slightly, if any [19, 35], but as they start to accumulate, their contributions to the λ_max_-shift become much more significant (Additional file 5: Table S3). More specifically, when the transmembrane III of AncAmphibian-359 is replaced by that of frog-423, the mutant pigment increases the λ_max_ to 410 nm (Δλ_max_ = 51 nm). This change is caused by V109A, E113D, L116V and S118T, but E113D in AncAmphibian-359 decreases its λ_max_ by 4 nm and V109A/L116V/S118T cause no λ_max_-shift at all [19]. This is not the whole story. Phylogenetic analyses strongly suggest that E113D occurred toward the end of frog-423 evolution; in that case, the four mutations contributed only 15 nm rather than 51 nm [35].

In addition to the SWS1 pigments, epistatic interactions have been found in RH1, RH1-like (RH2), SWS type 2 (SWS2) and M/LWS pigments as well [30, 36–39]. The most extensively studied mutations are forward (A292S) and reverse (S292A) changes in various pigments [17]. The λ_max_-shifts caused by A292S in RH1, SWS2 and M/LWS pigments range between −10 and 1 nm (e.g. [30, 40]), between −8 and −4 nm (e.g. [37, 38]) and −33 nm [39], respectively, whereas S292A in RH1, RH2, SWS1 and M/LWS pigments increase the λ_max_ by 4–12 nm [30], 7 nm [36], 0 nm [41] and 18–28 nm [40, 42], respectively. Hence, A292S and S292A mutants shift the λ_max_ by −33 ~ 1 and 0 ~ 28 nm, respectively, reflecting the varying levels of epistatic interactions between site 292 and its molecular background.

In studying possible molecular adaptation, it is common to “claim evidence of adaptive evolution based on computational analyses alone [43].” These sequence analyses are based basically on the assumption that “to detect positive Darwinian selection, it is necessary to show that the number of nonsynonymous substitutions per nonsynonymous site (dn) is significantly greater than that of synonymous substitutions per synonymous site (ds) [44, 45].” Indeed, such statistical methods have been used widely particularly in comparative genomics ([46, 47]; for SWS1 genes see [48]). However, given high rates of false-positives and false-negatives [30, 49], their reliabilities are questionable [47, 50–52]. Certainly, adaptive mutations identified using statistical methods generate biological hypotheses [52, 53], which must be tested eventually using experimental means [46, 47, 54].

We have seen that about 95 % of amino acid changes can be considered as “selectively neutral” changes. Currently, mutations at a total of 17 sites (positions 46, 49, 52, 86, 90, 91, 93, 97, 109, 113, 114, 116, 118, 179, 207, 256 and 277) can shift the λ_max_s of various SWS1 pigments, which seem to have contributed for adaptive evolution of various vertebrate species [13, 17, 35]. The average rates of nucleotide substitution at all positions of 300 codons of violet and UV pigments are both ~0.70 x 10^−9^/site/year; hence, as suspected, the effects of the small proportion of adaptive sites on the evolutionary rates are buried among those of the neutral changes (Table 4). However, when we consider the 16 codon sites that are involved in the λ_max_-shift, the rate of nucleotide substitution is significantly higher for violet pigments (1.73 x 10^−9^/site/year) than for the functionally unchanged UV pigments (0.70 x 10^−9^/site/year) (Table 4). This suggests that adaptive sites tend to be more susceptible for mutation accumulation [35].

**Table 4:** Rates of nucleotide substitutions for pairs of violet and UV opsin genes that are evaluated by comparing to those of distantly related third opsin genes

Visual pigment^a^				Evolutionary rate (10^−9^)	
Violet pigment	UV pigment	Third pigment	No. of codons^b^	Violet opsin gene	UV opsin gene
Scabbardfish-423	Tilapia-360	Goldfish-359	300	1.53 ± 0.13	1.42 ± 0.13
			16	3.54 ± 1.00	2.22 ± 0.73
Frog-423	Salamander-356	Zebra finch-359	300	0.76 ± 0.06^**^	0.40 ± 0.04^**^
			16	2.10 ± 0.55^**^	0.47 ± 0.19^**^
Chicken-415	Chameleon-359	Mouse-359	300	0.45 ± 0.05^**^	0.24 ± 0.03^**^
			16	1.30 ± 0.38^*^	0.29 ± 0.15^*^
zebra finch-359^c^	Chameleon-359	Mouse-359	300	0.30 ± 0.04	0.20 ± 0.03
			16	2.49 ± 0.65^**^	0 ± 0^**^
Human-414	Mouse-359	Mouse-419	300	0.57 ± 0.08	0.77 ± 0.10
			16	2.26 ± 0.78^*^	0.23 ± 0.22^*^
Bovine-438	Mouse-359	Elephant-419	300	0.70 ± 0.09	0.78 ± 0.10
			16	1.37 ± 0.58	0.84 ± 0.44
Squirrel-440	Mouse-359	Elephant-419	300	0.59 ± 0.09	0.65 ± 0.09
			16	0.70 ± 0.40	0.95 ± 0.47
Elephant-419	Mouse-359	Dunnart-363	300	0.52 ± 0.08	0.71 ± 0.09
			16	0.65 ± 0.37	0.65 ± 0.37
Wallaby-420	Dunnart-363	Mouse-359	300	0.78 ± 0.12	0.91 ± 0.13
			16	1.14 ± 0.64	0.67 ± 0.48
Average			300	0.69 ± 0.12	0.68 ± 0.13
			16	1.73 ± 0.32^**^	0.70 ± 0.22^**^

^a^The divergence times between scabbardfish and tilapia, between frog and salamander, between chicken and zebra finch, between human and mouse, between bovine and mouse, between squirrel and mouse, between elephant and mouse and between wallaby and dunnart are taken as 114, 298, 102, 98, 98. 98, 105 and 62 MY ago, respectively (www.timetree.org)

^b^Since F86 is missing from scabbarfish-423, 16 critical codon sites have been considered

^c^Zebra finch-359 is listed under violet pigment because it went through the transition of UV pigment → violet pigment → UV pigment during evolution [1]

* *P* < 0.05

** *P* < 0.01

The molecular analyses of phenotypic changes can also be found in such vertebrate systems as digestive enzymes secreted by the pancreas [55, 56], haemoglobins [57–60], steroid receptors [61–64] and olfactory receptors [65–67]. If we are serious about testing the statistical hypotheses of molecular adaptation, then this is an opportune time not only to explore these and other new genetic systems but also to improve the procedures to study the molecular mechanisms of phenotypic adaptation. Since phenotypic changes do not necessarily mean that they are adaptive, their adaptive nature must be established by linking phenotypic differences to differences in animals’ ecological and physiological environments [20, 21, 68]. Characterizing visual pigments considering HBN regions and establishing the one-to-one relationship between SWS1 pigment and UV/violet reception is a significant development in that direction.

## Conclusions

At present, molecular adaptations in vertebrates are studied almost always using comparative sequence analyses. These statistical results, however, provide only biological hypotheses and must be tested using experimental means. UV and violet reception, mediated by the SWS1 pigments with λ_max_s at ~360 and 395–440 nm, respectively, offer such an opportunity. The mechanisms of spectral tuning (or λ_max_-shift) and phenotypic adaptation of a present-day SWS1 pigment can be elucidated not only by engineering its ancestral pigment but also by using a new signature of protein structure, the A/B ratio. We can identify critical mutations that generated a present-day pigment by matching the λ_max_ and A/B ratio of an ancestral pigment with candidate mutations to that of the present-day pigment descended from it. Establishing the one-to-one relationship between SWS1 pigment and UV/violet reception is a significant development in actually testing statistical hypotheses of positive selection and also in understanding the mechanism of spectral tuning of the same pigment.

## Methods

### Reconstruction of ancestral SWS1 pigments

Previously, applying PAML [27] to a composite phylogenetic tree of 21 SWS1 pigments, the ancestral SWS1 pigments of jawed vertebrates (AncVertebrate), tetrapods (AncTetrapod), amphibians (AncAmphibian), amniotes (AncAmniote), Sauropsids (AncSauropsid), birds (AncBird) and Boreoeutheria (AncBoreotheria) were inferred and reconstructed [1].

Here, we considered the composite evolutionary tree of 33 representative SWS1 pigments based on molecular (e.g. www.timetree.org) and paleontological data (Fig. 1a): lamprey-358 (*Lamptera marinus*, U67123; λ_max =_ 358 [69]), goldfish-359 (*Carassius auratus auratus*, D85863; [70]), zebrafish-355 (*Danio rerio*, AB087810; [71]), scabbardfish-423 (*Lepidopus fitchi*, FJ443126; [26]), tilapia-360 (*Oreochromis niloticus*, AF191221; [72]), cichlid-Mzeb-368 (*Maylandia zebra*, AF191219; [73]), bfin killifish-355 (*Lucania goodie*, AY296735; [37]), medaka-356 (*Oryzias latipes*, AB223058; [74]), Pacific saury (*Cololabis saira*; KP099197), toothfish-369 (*Dissostichus mawsoni*, AY927651; [75]), lampfish-371 (*Stenobrachius leucepsarus*, FJ443127; [26]), frog-423 (*Xenopus laevis*, U23463; [19]), salamander-356 (*Ambystoma tigrinum*, AF038948; [76]), zebra finch-359 (*Taeniopygia guttata*, AF222331; [32]), canary-366 (*Serinus canaria*, AJ277922; [77]), budgerigar-363 (*Melopsittacus undulates*, Y11787; [33]), pigeon-393 (*Columba livia*, AF149234; [70]), chicken (*Gallus gallus*, M92039; [32]), chameleon-359 (*Anolis carolinensis*, AF134192; [78]), gecko-364 (*Gekko gecko*, AY024356; [79]), sunbeam-361 (*Xenopeltis unicolor*, FJ497234; [80]), human-414 (*Homo sapiens*, M13295; [18]), macaque-415 (*Macaca fascicularis*, AF158977; [81]), squirrel monkey-430 (*Saimiri sciureus*, U53875; [82]), marmoset-423 (*Callithrix jacchus*, L76201; [83]), bovine-438 (*Bos taurus*, U92557; [84]), mouse-359 (*Mus musculus*, U49720; [70]), rat-358 (*Rattus norvegicus*, U63972; [70]), squirrel-440 (*Sciurus carolinensis*, DQ302163; [85]), guinea pig-420 (*Cavia porcellus*, AY552608; [86]), elephant-419 (*Loxodonta Africana*, AY686753; [87]), wallaby-420 (*Macropus eugenii*, AY286017; [88]) and dunnart-363 (*Sminthopsis crassicaudata*, AY442173; [89]).

Using these sequences and those of RH1 pigment of bovine (*Bos taurus*, M21606), RH2 pigment of goldfish (*Carassius auratus*, L11865) and SWS2 pigment of zebrafish (*Danio rerio*, AB087809) as the outgroup, we inferred the amino acid sequences of SWS1 pigments at various nodes of the phylogenetic tree using PAML [27] using JTT and WAG substitution models.

When the amino acid sequences in the N and C termini of SWS1 pigments isolated from a wide range of vertebrate species are compared, the structures of the two segments differ significantly by including insertions and deletions. To evaluate the effects of these variable N and C termini on the λ_max_-shift, the N terminus (amino acids between sites 1 and 30) and the C terminus (those between sites 313 and 348) of the SWS1 pigment of mouse-359 were replaced by those of goldfish (*Carassius auratus*), chameleon (*Anolis carolinensis*) and human. The respective chimeric pigments had λ_max_ values of 360, 359 and 360 nm, showing that the highly polymorphic amino acids at the two termini do not modify the λ_max_ values of SWS1 pigments [1]. Hence, the previously reconstructed ancestral pigments contained the identical N and C termini of the chameleon SWS1 pigment plus the ancestral pigment-specific internal segment between sites 31 and 312 [1]. Following the same strategy, we reconstructed several ancestral pigments by introducing the necessary amino acid changes into the internal segment (sites 31 and 312) in the pMT5 containing the N- and C-termini of the chameleon-359.

All mutant opsins were generated by using QuickChange site-directed mutagenesis kits (Stratagene, La Jolla, CA). To rule out spurious mutations, the DNA fragment was sequenced by cycle sequencing reactions using the Sequitherm Excel II long-read kits (Epicentre Technologies, Madison, WI) with dye-labeled M13 forward and reverse primers. Reactions were run on a LI-COR (Lincoln, NE) 4300LD automated DNA sequencer.

### Mutagenesis genesis data of ancestral and present-day pigments

Previously, the λ_max_s of ancestral and present-day pigments as well as mutant pigments (mutations in subscripts) have been obtained by various authors: AncSauropsid-360, AncBird-393, AncBird^*^-393, AncSauropsid-360_F86S_, AncBird^*^-393_S86C_, AncBird^*^-393_S90C_, AncSauropsid-393_A114G_, AncSauropsid-360_F49V/F86S/L116V/S118A_, AncBird-393_V49F/S86F/V116L/A118S_ [1], AncBoreotheria-360, AncBoreotheria-360_F86L_ [35], bfin killifish-354 [37], goldfish-360, Pigeon-393, mouse-359 [70], lampfish-371, scabbardfish-423, AncVertebrate-361_del (F86)_, lampfish-371_del (F86)_, bfin killifish-355_del (F86)_, scabbardfish-423_ins (F86)_ [26], chameleon-359 [78], zebra finch-359, zebra finch-359_C90S_, chicken-415_S90C_, pigeon-393_S90C_ [32, 90], budgerigar-363, budgerigar-363_C90S_ [33], dunnart-363 [89], frog-423, AncAmphibian-359_F86M_, AncAmphibian-359_E113D_, AncAmphibian-359_F86M/V91I/T93P/V109A/E113D/L116V/S118T_, frog-423_M86F/I91V/P93T/A109V/D113E/V116L/T118S_ [19], chicken-413 [91], human-414, mouse -359_S90C_, mouse-359_A114G_, human-414_T46F/L49F/F52T/L86F/P93T/G114A/T118S_ [18], bovine-438, mouse -359_F86Y_, bovine-438_Y86F_, bovine-438_S90C_, mouse-359_T93I_, mouse-359_F86Y/T93I_, bovine-438_Y86F/I93T_, bovine-438_Y86F/I93T/T97S_ [84], squirrel-440, squirrel-440_Y86F_ [85], elephant-419, elephant-419_S86F_, elephant-419_I93T_, elephant-419_S86F/I93T_, elephant-419_I93T/V116L_, elephant-419_S86F/V116L_ [87], wallaby-420 [88], bovine-438_Y86F_, goldfish-360_F86Y_ [92], frog-423_S90C_ [93] and frog-423_D113E_ [94]. Note that two different Δλ_max_ values have been obtained from a dark spectrum [84] or from a dark–light spectrum [92]. In our comparison, we considered the former results simply because it is a more direct measurement.

We also added the λ_max_s of AncVertebrate-361, AncEuteleost-364, AncTetrapod-359, AncAmniote-359, AncAmphibian-359, AncMammal-359, AncEutheria-360, AncEuteleost-364_del (F86)_, human-414_L86F_, frog-423_M86F_, AncEutheria-360_F86S_, AncBird-393_S86F_, AncMammal-359_F86Y_, wallaby-420_Y86F_, AncBoreotheria-360_F86Y_, AncBird-393_S86C_, zebra finch-359_C86S_, AncEutheria-360_T93I_, AncEutheria-360_T93I_, bovine-438_I93T_, bovine-438_Y86F/I93T_, AncEutheria-360_F86S/T93I_, AncBoreotheia-360_T93I/S97T_, bovine-438_I93T/T97S_, AncEutheria-360_T93I/L116V_, AncEutheria-360_F86S/L116V_, AncEutheria-360_F86S/T93I/L116V_, elephant-419_S86F/I93T/V116L_, AncBoreotheia-360_F86Y/T93I/S97T_ and AncBoreotheria-360_F46T/F49L/T52F/F86L/T93P/A114G/S118T._

### The *in vitro* assay

Ancestral and other mutant opsins were expressed in COS1 cells by transient transfection [28]. The COS1 cell is a simian cell that is permissive for SV 40 replication and supports the replication of a recombinant DNA molecule containing a SV40 origin of replication that requires no ethical approvals or permissions. The pigments were regenerated by incubating the opsins with 11-*cis*-retinal (a gift from Dr. Rosalie K. Crouch at Storm Eye Institute, Medical University of South Carolina and National Eye Institutes) and were purified using immobilized 1D4 (The Culture Center, Minneapolis, MN) in buffer W1 (50 mM N-(2-hydroxyethyl) piperazine-N’-2-ethanesulfonic acid (HEPES) (pH 6.6), 140 mM NaCl, 3 mM MgCl_2_, 20 % (w/v) glycerol and 0.1 % dodecyl maltoside). UV visible spectra were recorded at 20 °C using a Hitachi U-3000 dual beam spectrophotometer. Visual pigments were bleached for 3 min using a 60 W standard light bulb equipped with a Kodak Wratten #3 filter at a distance of 20 cm. Data were analyzed using Sigmaplot software (Jandel Scientific, San Rafael, CA).

### Linear models

To estimate the effects of individual and epistatic interactions of amino acid changes (θs) on the λ_max_-shift, we used linear statistical models, where the λ_max_ of each visual pigment is expressed as a function of the λ_max_ of AncEutheria-360 (or elephant-419) [87] and the effects of the appropriate single and multiple amino acid changes on the λ_max_-shift [95].

### Modelling of protein structures

The initial structures of contemporary, ancestral and mutant pigments were obtained from their amino acid sequences with homology modelling (SWISS MODEL [34]) by taking the structure of bovine rhodopsin (pdb code: 1U19) as a template, which are called SWISS models. SWISS model structures of five representative pigments were further refined by 1) adding the missing hydrogen atoms and 2) full AMBER96 (http://ambermd.org) [24, 25] geometry optimizations, which are called AMBER models. In this protein modelling, we excluded the 11-*cis*-retinal not only because such protein structures can reveal whether or not the retinal can actually fit into the retinal-binding pocket but also because widely accessible protein modelling methods exclude the 11-*cis*-retinal. We also performed multivariable linear regression analysis between λ_max_ values (dependent variable) and the structural parameters A, B, and A/B derived from the AMBER models (independent variable) [95].

### Sequence analyses

The proportion (p) of different nucleotides was computed for each pairwise comparison and the number (d) of nucleotide substitutions per site was estimated from d = − (3/4) ln [1 – (4/3)p] [96]. For a set of violet opsin gene (A), UV opsin gene (B) and evolutionarily more distantly related third gene (C), the numbers of nucleotide substitutions per site between genes A and B (d_AB_), between genes A and C (d_AC_) and between genes B and C (d_BC_) were evaluated, and then the branch lengths for genes A and B after their divergence are determined by (1/2) (d_AB_ + d_AC_ – d_BC_) and (1/2) (d_AB_ - d_AC_ + d_BC_), respectively. The rates of nucleotide substitution for opsin genes A and B were evaluated by dividing their branch lengths by appropriate divergence times that were estimated from the timetree of life (www.timetree.org). The divergence times between scabbardfish-423 and tilapia-360, between frog-423 and salamander-356, between chicken-415 and zebra finch-359, between human-414 and mouse-359, between bovine-438 and mouse-359, between squirrel-440 and mouse-359, between elephant-419 and mouse-359 and between wallaby-420 and dunnart-363 are taken as 114, 298, 102, 98, 98. 98, 105 and 62 MY ago, respectively (www.timetree.org). Since F86 is missing from scabbardfish-423, 16 critical codon sites have been considered. Note that zebra finch-359 is listed under violet pigment because it went through the transition of UV pigment → violet pigment → UV pigment during evolution [1]. Standard errors for the evolutionary rates were estimated from [9p(1-p)/{(3–4p)^2^n}]^1/2^, where n is the number of nucleotide sites considered.

### Ethics statement

All DNA sequence data were taken from publically available sources and no animal experiments were conducted in this study

### Availability of supporting data

The authors confirm that all data underlying the findings are fully available without restriction. All relevant data are within the Methods and in the Additional files section.

## Additional files

Additional file 1: Table S1. Amino acids of AncVertebrate-361 with PP < 0.95 (in parentheses) inferred using PAML with JTT and WAG models. (DOCX 36 kb) Additional file 2: Figure S1. The amino acid sequences of 10 ancestral pigments. Ancestral pigments are reconstructed by introducing all different amino acids between sites 31 and 312 (see Methods). Amino acids with PP < 0.95 are indicated by bold italics. Four critical amino acids that caused the evolution of AncBird-393 from AncSauropsid-360 are indicated by black boxes. (PDF 33 kb) Additional file 3: Figure S2. The absorption spectra of seven ancestral pigments. (PDF 451 kb) Additional file 4: Table S2. The λ_max_s and A/B ratios of HBNs of ancestral and present-day pigments. (DOCX 43 kb) Additional file 5: Table S3. Variable λ_max_-shifts and A/B ratios caused by various mutations. (DOCX 37 kb)

## Abbreviations

Å: Angstrom or 10^−10^ m
AMBER: Assisted model building with energy refinement software package
AncAmniote: The ancestral pigment of Amniotes
AncAmphibian: The ancestral pigment of Amphibians
AncBird: The ancestral pigment of Aves
AncBird*-393: AncSauropsid-360 with mutations F49V/F86S/L116V/S118A
AncBoreotheria: The ancestral pigment of Boreoeutherians
AncEuteleost: The ancestral pigment of Euteleosts, AncEutheria, the ancestral pigment of Eutherians
AncMammal: The ancestral pigment of the common ancestor of Placentals and Marsupials
AncSauropsid: The ancestral pigment of Sauropsids
AncTetrapod: The ancestral pigment of Tetrapods
AncVertebrate: The ancestral pigment of jawed vertebrates
C-terminus: COOH-terminus
d(A/B): The A/B ratio of an ancestral pigment subtracted from that of a mutant pigment
d(λ_max_): the λ_max_ of an ancestral pigment subtracted from that of a mutant pigment
Δλ_max_: λ_max_-shift
HBN: Hydrogen-bond network
JTT: Jones-Taylor-Thornton
λ_elephant_: the λ_max_ of elephant-419
λ_max_: The wavelength of maximal absorption
M/LWS: Middle and long wavelength-sensitive
N-terminus: NH_2_-terminus
PAML: Phylogenetic analysis by maximum likelihood
PP: Posterior probability
PSBR: Pigments with protonated Schiff base nitrogen-linked retinals
RH1 pigment: Rhodopsin
SBR: Pigments with unprotonated Schiff base nitrogen-linked retinals
SB: Schiff base
SWISS-MODEL: A fully automated protein structure homology-modelling server
SWS1: short wavelength-sensitive type 1
SWS2: SWS type 2
UV: Ultraviolet
θ_i_: the epistatic effect of mutation i on the λ_max_-shift
WAG: Whelan and Goldman
