# Supplementary material for: A simple method for studying the molecular mechanisms of ultraviolet and violet reception in vertebrates
Source: BMC Evol Biol. 2016 Mar 22;16:64. doi: 10.1186/s12862-016-0637-9 (PMC4802639; doi:10.1186/s12862-016-0637-9)
Supplement: Additional file 1: Table S1. — Amino acids of AncVertebrate-361 with PP < 0.95 (in parentheses) inferred using PAML with JTT and WAG models. (DOCX 36 kb) [file 12862_2016_637_MOESM1_ESM.docx]

**Table S1.** Amino acids of AncVertebrate-361 with PP < 0.95 (in parentheses) inferred using PAML with JTT and WAG models.

| Site | JTT | | WAG | | Site | JTT | | WAG | |
| --- | --- | --- | --- | --- | --- | --- | --- | --- | --- |
| 22 | G (0.95) | S (0.05) | G (0.93) | S (0.07) | 166 | G (0.77) | S (0.21) | G (0.68) | S (0.30) |
| 25 | D (0.95) | E (0.05) | D (0.94) | E (0.06) | 167 | V (0.94) | C (0.06) | V (0.88) | C (0.12) |
| 34 | K (0.71) | A (0.15) | K (0.89) | A (0.08) | 168 | A (0.94) | S (0.06) | A (0.95) | S (0.05) |
| 41 | A (0.92) | T (0.07) | A (0.96) | T (0.94) | 169 | I (0.88) | L (0.08) | I (0.83) | L (0.13) |
| 60 | I (0.53) | V (0.46) | I (0.56) |  (0.42) | 195 | G (0.93) | N (0.06) | G (0.91) | N (0.09) |
| 85 | I (0.68) |  (0.20) | I (0.67) |  (0.17) | 199 | K (0.73) | N (0.18) | K (0.61) | N (0.28) |
| 88 | I (0.87) | L (0.06) | I (0.82) | L (0.10) | 209 | V (0.83) | I (0.17) | V (0.86) | I (0.14) |
| 91 | V (0.91) | I (0.09) | V (0.91) | I (0.09) | 213 | I (0.87) | V (0.12) | I (0.92) | V (0.08) |
| 97 | S (0.94) | A (0.06) | S (0.95) | A (0.05) | 214 | V (0.90) | I (0.09) | V (0.86) | I (0.13) |
| 99 | T (0.43) | S (0.34) | T (0.43) | S (0.34) | 224 | S (0.82) | G (0.17) | S (0.84) | G (0.15) |
| 105 | F (0.59) | L (0.41) | F (0.58) | L (0.42) | 229 | A (0.92) | T (0.08) | A (0.95) | T (0.05) |
| 107 | R (0.48) | H (0.26) | R (0.51) | P (0.28) | 242 | S (0.93) | T (0.07) | S (0.94) | T (0.06) |
| 109 | V (0.59) | I (0.37) | V (0.48) | I (0.46) | 263 | T (0.45) | L (0.27) | T (0.42) | L (0.35) |
| 116 | L (0.65) | M (0.29) | M (0.51) | L (0.41) | 271 | L (0.79) | M (0.18) | L (0.82) | M (0.16) |
| 119 | V (0.53) | I (0.33) | I (0.40) | V (0.40) | 273 | M (0.75) | V (0.15) | M (0.75) | V (0.10) |
| 155 | V (0.54) | A (0.24) | A (0.44) | M (0.29) | 280 | D (0.93) | N (0.07) | D (0.90) | N (0.10) |
| 158 | A (0.70) | V (0.23) | A (0.86) | V (0.07) | 283 | L (0.68) | I (0.27) | L (0.66) | I (0.29) |
| 159 | L (0.56) | F (0.38) | L (0.54) | F (0.40) | 304 | L (0.89) | I (0.17) | L (0.80) | I (0.19) |
| 162 | V (0.57) | I (0.38) | V (0.59) | I (0.37) | 307 | S (0.95) | A (0.04) | S (0.94) | A (0.05) |
