# Supplementary material for: A simple method for studying the molecular mechanisms of ultraviolet and violet reception in vertebrates
Source: BMC Evol Biol. 2016 Mar 22;16:64. doi: 10.1186/s12862-016-0637-9 (PMC4802639; doi:10.1186/s12862-016-0637-9)
Supplement: Additional file 2: Figure S1. — The amino acid sequences of 10 ancestral pigments. Ancestral pigments are reconstructed by introducing all different amino acids between sites 31 and 312 (see Methods). Amino acids with PP < 0.95 are indicated by bold italics. Four critical amino acids that caused the evolution of AncBird-393 from AncSauropsid-360 are indicated by black boxes. (PDF 33 kb) [file 12862_2016_637_MOESM2_ESM.pdf]

```
                       30        40        50        60        70        80        90
                        .         .         .         .         .         .         .
AncVertebrate  GPWDGPQYHIAPAWAFYLQAAFMGFVFFVGTPLNAIVLIVTIKYKKLRQPLNYILVNISLAGFIFCIFS
AncEuteleost   SPFEGPQYHLAPKWAFYLQAAFMGFVFFVGTPLNAIVLFVTMKYKKLRQPLNYILVNISLAGFIFVTFS
AncTetrapod    GPWDGPQYHIAPAWAFYLQTAFMGFVFFVGTPLNAIVLIVTIKYKKLRQPLNYILVNISLAGFIFCIFS
AncAmphibian   GPWDGPQYHIAPAWAFYLQTAFMGFVFFVGTPLNAIVLIVTIKYKKLRQPLNYILVNISLAGFIFCIFS
AncAmniote     GPWDGPQYHIAPAWAFYLQTAFMGFVFFVGTPLNAIVLIVTIKYKKLRQPLNYILVNISLAGFIFCIFS
AncSauropsid   GPWDGPQYHIAPAWAFYLQTAFMGFVFFVGTPLNAIVLIVTIKYKKLRQPLNYILVNISFAGFIFCIFS
AncBird        GPWDGPQYHIAPPWAFYLQTAFMGFVFVVGTPLNAIVLVVTIKYKKLRQPLNYILVNISFSGFISCIFS
AncMammal      GPWDGPQYHIAPAWAFHLQTAFMGFVFFVGTPLNAIVLIATLRYKKLRQPLNYILVNISLAGFIFCIFS
AncEutheria    GPWDGPQYHIAPVWAFHLQAAFMGFVFFVGTPLNAIVLVATLRYKKLRQPLNYILVNISLGGFLFCIFS
AncBoreotheria GPWDGPQYHIAPVWAFHLQAAFMGFVFFVGTPLNAIVLVATLRYKKLRQPLNYILVNVSLGGFLFCIFS
                       100       110       120       130       140       150       160
                        .         .         .         .         .         .         .
AncVertebrate  VSTVFVSSTQGYFFFGRTVCALEAFLGSVAGLVTGWSLAFLAFERYIVICKPFGNFRFGSKHALVAVALT
AncEuteleost   VSQVFVSSTRGYFFLGHTLCALEAAMGSIAGLVTGWSLAVLAFERYVVICKPFGSFKFGSNHALAAVAFT
AncTetrapod    VFTVFVSSSQGYFFFGRTVCALEAFLGSVAGLVTGWSLAFLAFERYIVICKPFGNFRFSSKHALMVVVAT
AncAmphibian   VFTVFVSSSQGYFFFGRTVCALEAFLGSVAGLVTGWSLAFLAFERYIVICKPMGNFRFSSKHALMVVVAT
AncAmniote     VFTVFVSSSQGYFFFGRHVCALEAFLGSVAGLVTGWSLAFLAFERYIVICKPFGNFRFNSKHALMVVVAT
AncSauropsid   VFTVFVSSSQGYFFFGRHVCALEAFLGSVAGLVTGWSLAFLAFERYIVICKPFGNFRFNSKHALLVVVAT
AncBird        VFTVFVSSSQGYFVFGKHVCALEAFVGATGGLVTGWSLAFLAFERYIVICKPFGNFRFNSKHALLVVVAT
AncMammal      VFTVFISSSQGYFVFGRHVCALEAFLGSVAGLVTGWSLAFLAFERYIVICKPFGNFRFNSKHALMVVLAT
AncEutheria    VFTVFISSCQGYFVFGRHVCALEAFLGSVAGLVTGWSLAFLAFERYIVICKPFGNFRFSSKHALMVVLAT
AncBoreotheria VFTVFIASCHGYFVFGRHVCALEAFLGSVAGLVTGWSLAFLAFERYIVICKPFGNFRFSSKHALMVVLAT
                       170       180       190       200       210       220       230
                        .         .         .         .         .         .         .
AncVertebrate  WVIGIGVAIPPFFGWSRYIPEGLQCSCGPDWYTVGTKYKSEYYTYFLFVFCFIVPLSIIIFSYSQLLGAL
AncEuteleost   WIIGIGCATPPFFGWSRYIPEGLGCSCGPDWYTKNEEYNSESYTYFLLVTCFIMPLTIIIFSYSQLLGAL
AncTetrapod    WVIGIGVSIPPFFGWSRYIPEGLQCSCGPDWYTVGTKYKSEYYTWFLFIFCFIVPLSLICFSYSQLLGAL
AncAmphibian   WVIGIGVSIPPFFGWSRYIPEGLQCSCGPDWYTVGTKYKSEYYTWFLFIFCFIIPLSLICFSYSQLLGAL
AncAmniote     WVIGIGVSIPPFFGWSRYIPEGLQCSCGPDWYTVGTKYKSEYYTWFLFIFCFIVPLSLICFSYSQLLGAL
AncSauropsid   WVIGIGVSIPPFFGWSRYIPEGLQCSCGPDWYTVGTKYKSEYYTWFLFIFCFIVPLSLIIFSYSQLLGAL
AncBird        WVIGIGVAIPPFFGWSRYIPEGLQCSCGPDWYTVGTKYKSEYYTWFLFIFCFIVPLSLIIFSYSQLLSAL
AncMammal      WVIGIGVSIPPFFGWSRYIPEGLQCSCGPDWYTVGTKYRSEYYTWFLFIFCFIVPLSLICFSYSQLLGAL
AncEutheria    WTIGIGVSIPPFFGWSRFIPEGLQCSCGPDWYTVGTKYRSEYYTWFLFIFCFIVPLSLICFSYSQLLGAL
AncBoreotheria WTIGIGVSIPPFFGWSRFIPEGLQCSCGPDWYTVGTKYRSEYYTWFLFIFCFIVPLSLICFSYSQLLRAL
                       240       250       260       270       280       290       300
                        .         .         .         .         .         .         .
AncVertebrate  RAVAAQQQESASTQKAEREVSRMVIVMVGSFCTCYVPYAALAMYMVNNRDHNLDLRLVTIPAFFSKSSCV
AncEuteleost   RAVAAQQAESASTQKAEKEVSRMVIVMVGSFVTCYGPYAITALYFANSTDPNKDYRLVTIPAFFSKSSCV
AncTetrapod    RAVAAQQQESATTQKAEREVSRMVIVMVGSFCLCYVPYAALAMYMVNNRNHGLDLRLVTIPAFFSKSSCV
AncAmphibian   RAVAAQQQESATTQKAEREVSRMVIVMVGSFCLCYVPYAAMAMYMVNNRNHGLDLRLVTIPAFFSKSSCV
AncAmniote     RAVAAQQQESATTQKAEREVSRMVVVMVGSFCLCYVPYAALAMYMVNNRNHGLDLRLVTIPAFFSKSSCV
AncSauropsid   RAVAAQQQESATTQKAEREVSRMVVVMVGSFCLCYVPYAALAMYMVNNRNHGLDLRLVTIPAFFSKSSCV
AncBird        RAVAAQQQESATTQKAEREVSRMVVVMVGSFCLCYVPYAALAMYMVNNRNHGLDLRLVTIPAFFSKSSCV
AncMammal      RAVAAQQQESATTQKAEREVSRMVVVMVGSFCLCYVPYAALAMYMVNNRNHGLDLRLVTIPAFFSKSSCV
AncEutheria    RAVAAQQQESATTQKAEREVSRMVVVMVGSFCLCYVPYAALAMYMVNNRNHGLDLRLVTIPAFFSKSSCV
AncBoreotheria RAVAAQQQESATTQKAEREVSRMVVVMVGSFCLCYVPYAALAMYMVNNRNHGLDLRLVTIPAFFSKSSCV
                       310       320
                        .         .
AncVertebrate  YNPLIYSFMNKQFRACIMETVC
AncEuteleost   YNPLIYAFMNKQFNACIMETVF
AncTetrapod    YNPIIYSFMNKQFRACIMETVC
AncAmphibian   YNPIIYSFMNKQFRACIMETVC
AncAmniote     YNPIIYCFMNKQFRACIMETVC
AncSauropsid   YNPIIYCFMNKQFRACIMETVC
AncBird        YNPIIYCFMNKQFRACIMETVC
AncMammal      YNPIIYCFMNKQFRACIMEMVC
AncEutheria    YNPIIYCFMNKQFRACIMEMVC
AncBoreotheria YNPIIYCFMNKQFRACIMEMVC
```
