# Supplementary figures and images for: A simple method for studying the molecular mechanisms of ultraviolet and violet reception in vertebrates
Source: BMC Evol Biol. 2016 Mar 22;16:64. doi: 10.1186/s12862-016-0637-9 (PMC4802639; doi:10.1186/s12862-016-0637-9)

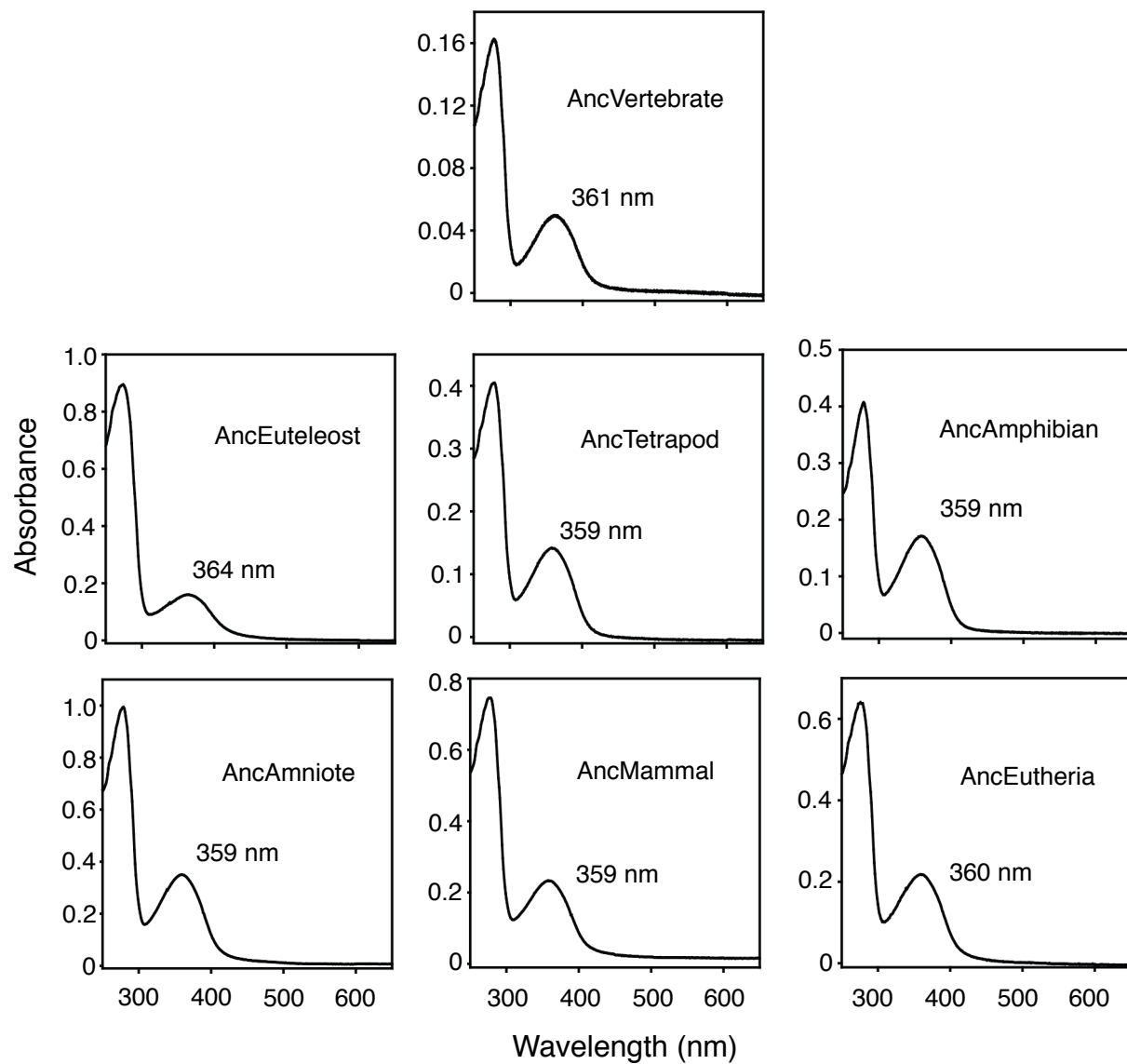

Supplement: Additional file 3: Figure S2. — The absorption spectra of seven ancestral pigments. (PDF 451 kb) [file 12862_2016_637_MOESM3_ESM.pdf]
