# Supplementary material for: A simple method for studying the molecular mechanisms of ultraviolet and violet reception in vertebrates
Source: BMC Evol Biol. 2016 Mar 22;16:64. doi: 10.1186/s12862-016-0637-9 (PMC4802639; doi:10.1186/s12862-016-0637-9)
Supplement: Additional file 4: Table S2. — The λmaxs and A/B ratios of HBNs of ancestral and present-day pigments. (DOCX 43 kb) [file 12862_2016_637_MOESM4_ESM.docx]

**Table S2.** The λ_max_s and A/B ratios of HBNs of ancestral and present-day pigments.

| Pigment_mutation_ | Δλ_max_  (nm) | A (Å^2^) | B (Å^2^) | A/B |
| --- | --- | --- | --- | --- |
|  |  |  |  |  |
| AncVertebrate-361_del (F86)_ | 19^*^ | 26.369 | 75.119 | 0.542 → 0.351 |
| AncEuteleost-364_del (F86)_ | ND | - | - | - |
| lampfish-371_del (F86)_ | 37^*^ | 27.119 | 75.783 | 0.542 → 0.358 |
| bfin killifish-355_del (F86)_ | 58^*^ | 28.469 | 74.395 | 0.577 → 0.383 |
| scabbardfish-423_ins (F86)_ | -60^*^ | 32.069 | 59.102 | 0.355 → 0.543 |
|  |  |  |  |  |
| AncBoreotheria-360_F86L_ | 0^*^ | 31.804 | 60.795 | 0.577 → 0.523 |
| human-414_L86F_ | -32 | 31.422 | 57.891 | 0.519 → 0.543 |
|  |  |  |  |  |
| AncAmphibian-359_F86M_ | 2^*^ | 32.027 | 66.406 | 0.541 → 0.482 |
| frog-423_M86F_ | -25 | 29.129 | 58.375 | 0.439 → 0.499 |
|  |  |  |  |  |
| AncSauropsid-360_F86S_ | 17^*^ | 32.027 | 69.133 | 0.542 → 0.463 |
| AncEutheria-360_F86S_ | 14 | 32.027 | 69.133 | 0.542 → 0.463 |
| AncBird-393_S86F_ | -38 | 33.354 | 57.763 | 0.493 → 0.577 |
| elephant-419_S86F_ | -52^*^ | 31.153 | 58.389 | 0.445 → 0.533 |
|  |  |  |  |  |
| AncMammal-359_F86Y_ | 57 | 31.975 | 61.390 | 0.542 → 0.521 |
| wallaby-420_Y86F_ | -59 | 32.086 | 59.101 | 0.522 → 0.543 |
|  |  |  |  |  |
| AncBoreotheria-360_F86Y_ | 48 | 31.804 | 59.985 | 0.548 → 0.530 |
| mouse -359_F86Y_ | 66^*^ | 31.717 | 59.717 | 0.547 → 0.531 |
| bovine-438_Y86F_^§^ | -71^*^ | 31.918 | 60.157 | 0.510 → 0.530 |
| bovine-438_Y86F_^§^ | -75^*^ | 31.918 | 60.157 | 0.510 → 0.530 |
| squirrel-440_Y86F_ | -76^*^ | 31.579 | 58.042 | 0.526 → 0.544 |
| goldfish-360_F86Y_ | 60^*^ | 32.133 | 61.712 | 0.543 → 0.521 |
|  |  |  |  |  |
| AncBird-393_S86C_ | -7 | 33.411 | 67.996 | 0.493 → 0.491 |
| AncBird^*^-393_S86C_ | -27^*^ | 33.396 | 68.828 | 0.492 → 0.485 |
| zebra finch-359_C86S_ | 1 | 35.008 | 66.155 | 0.535 → 0.529 |
|  |  |  |  |  |
| frog-423_S90C_ | -37* | 30.556 | 64.106 | 0.446 → 0.477 |
| AncBird^*^-393_S90C_ | -33* | 35.021 | 66.351 | 0.492 → 0.528 |
| chicken-415_S90C_ | -46* | 32.223 | 67.988 | 0.439 → 0.474 |
| pigeon-393_S90C_ | -34* | 34.997 | 66.258 | 0.492 → 0.528 |
| bovine-438_S90C_ | -7* | 33.470 | 61.036 | 0.510 → 0.548 |
| mouse-359_S90C_ | 0* | 33.347 | 56.418 | 0.547 → 0.591 |
| zebra finch-359_C90S_ | 38* | 33.378 | 68.009 | 0.577 → 0.491 |
| budgerigar-363_C90S_ | 35* | 33.486 | 67.103 | 0.535 → 0.499 |
|  |  |  |  |  |
| AncEutheria-360_T93I_ | 0 | 32.021 | 59.091 | 0.542 → 0.542 |
| AncBoreotheria-360_T93I_ | -4 | 31.679 | 58.019 | 0.548 → 0.546 |
| mouse-359_T93I_ | 0* | 31.679 | 58.019 | 0.547 → 0.546 |
| elephant-419_I93T_ | -6* | 30.565 | 70.933 | 0.445 → 0.431 |
| bovine-438_I93T_ | -22 | 31.918 | 62.572 | 0.510 → 0.510 |
|  |  |  |  |  |
| AncAmphibian-359_E113D_ | -4* | 29.878 | 59.093 | 0.541 → 0.506 |
| frog-423_D113E_ | -12* | 30.409 | 65.785 | 0.439 → 0.462 |
|  |  |  |  |  |
| mouse-359_A114G_ | 0* | 31.783 | 58.019 | 0.547 → 0.548 |
| AncSauropsid-393_A114G_ | 5* | 32.067 | 59.103 | 0.542 → 0.542 |
|  |  |  |  |  |
| AncEutheria-360_F86S/T93I_ | 55 | 32.020 | 69.551 | 0.542 → 0.460 |
| elephant-419_S86F/I93T_ | -60* | 30.650 | 58.587 | 0.445 → 0.523 |
|  |  |  |  |  |
| AncBoreotheia-360_T93I/S97T_ | 1 | 31.773 | 58.056 | 0.548 → 0.547 |
| bovine-438_I93T/T97S_ | -39 | 31.918 | 62.572 | 0.510 → 0.510 |
|  |  |  |  |  |
| AncEutheria-360_T93I/L116V_ | 0 | 32.115 | 59.045 | 0.542 → 0.544 |
| elephant-419_I93T/V116L_ | -8* | 31.691 | 69.770 | 0.445 → 0.454 |
|  |  |  |  |  |
| AncEutheria-360_F86S/L116V_ | 38 | 32.066 | 69.082 | 0.542 → 0.464 |
| elephant-419_S86F/V116L_ | -59* | 31.779 | 58.038 | 0.445 → 0.547 |
|  |  |  |  |  |
| mouse-359_F86Y/T93I_ | 66* | 31.679 | 60.028 | 0.547 → 0.528 |
| bovine-438_Y86F/I93T_ | -68* | 31.877 | 60.166 | 0.510 → 0.530 |
|  |  |  |  |  |
| AncEutheria-360_F86S/T93I/L116V_ | 50 | 32.066 | 69.118 | 0.542 → 0.478 |
| elephant-419_S86F/I93T/V116L_ | -59 | 31.779 | 58.038 | 0.445 → 0.547 |
|  |  |  |  |  |
| AncBoreotheia-360_F86Y/T93I/S97T_ | 48 | 31.773 | 59.993 | 0.548 → 0.530 |
| bovine-438_Y86F/I93T/T97S_ | -79* | 31.952 | 60.162 | 0.510 → 0.531 |
|  |  |  |  |  |
| AncSauropsid -360  ^F49V/F86S/L116V/S118A^ | 33* | 33.396 | 67.890 | 0.542 → 0.492 |
| AncBird-393_V49F/S86F/V116L/A118S_ | -33* | 32.088 | 59.109 | 0.493 → 0.543 |
|  |  |  |  |  |
| AncBoreotheria-360  ^F46T/F49L/T52F/F86L/T93P/A114G/S118T^ | 52 | 31.422 | 60.574 | 0.548 → 0.519 |
| human-414  ^T46F/L49F/F52T/L86F/P93T/G114A/T118S^ | -54* | 31.765 | 58.047 | 0.519 → 0.547 |
|  |  |  |  |  |
| AncAmphibian-359  ^F86M/V91I/T93P/V109A/E113D/L116V/S118T^ | 62* | 28.839 | 65.390 | 0.544 → 0.441 |
| frog-423  ^M86F/I91V/P93T/A109V/D113E/V116L/T118S^ | -68* | 32.186 | 59.033 | 0.439 → 0.545 |

*For the data source, see Materials and methods. ^§^The first and second values were

obtained using dark and dark-light spectra. AncBird*-393 is identical to

AncSauropsid-360 with F49V/F86S/L116V/S118A. The A/B ratios for the pigments

with F86 deletion are evaluated by considering amino acids at site 91 in place of 86.
