# Supplementary material for: A simple method for studying the molecular mechanisms of ultraviolet and violet reception in vertebrates
Source: BMC Evol Biol. 2016 Mar 22;16:64. doi: 10.1186/s12862-016-0637-9 (PMC4802639; doi:10.1186/s12862-016-0637-9)
Supplement: Additional file 5: Table S3. — Variable λmax-shifts and A/B ratios caused by various mutations. (DOCX 37 kb) [file 12862_2016_637_MOESM5_ESM.docx]

**Table S3.** Variable λ_max_-shifts and A/B ratios caused by various mutations.

| Pigment | λ_max_ (nm) | A (Å^2^) | B (Å^2^) | A/B |
| --- | --- | --- | --- | --- |
| Group 1 |  |  |  |  |
| AncVertebrate-361 | 361 | 32.057 | 59.109 | 0.542 |
| AncEuteleost-364 | 364 | 32.069 | 59.158 | 0.542 |
| AncTetrapod-359 | 359 | 32.040 | 59.040 | 0.543 |
| AncAmniote-359 | 359 | 32.040 | 59.040 | 0.543 |
| AncAmphibian-359 | 359 | 32.121 | 59.093 | 0.544 |
| AncSauropsid-360 | 360* | 32.027 | 59.101 | 0.542 |
| AncMammal-359 | 359 | 32.157 | 59.032 | 0.542 |
| AncEutheria-360 | 360 | 32.027 | 59.101 | 0.542 |
| AncBoreotheria-360 | 360* | 31.804 | 58.012 | 0.548 |
| bfin killifish-354 | 354* | 33.331 | 57.746 | 0.577 |
| goldfish-360 | 359* | 32.091 | 59.112 | 0.543 |
| lampfish-371 | 371* | 32.141 | 59.266 | 0.542 |
| chameleon-359 | 359* | 32.186 | 59.032 | 0.545 |
| zebra finch-359 | 359* | 36.937 | 64.031 | 0.577 |
| budgerigar-363 | 363* | 35.072 | 65.602 | 0.535 |
| mouse-359 | 359* | 31.773 | 58.056 | 0.547 |
| dunnart-363 | 363* | 32.057 | 59.101 | 0.542 |
| Average |  | 32.584 (± 0.332) | 59.567 (± 0.496) | 0.547 (± 0.003) |
| Conf. interval (95%) |  | 31.93 - 33.23 | 58.59 - 60.54 | 0.54 - 0.55 |
|  |  |  |  |  |
| Group 2 |  |  |  |  |
| AncBird-393 | 393* | 33.396 | 67.758 | 0.493 |
| AncBird^*^-393^†^ | 393* | 33.40 | 67.89 | 0.492 |
| Pigeon-393 | 393* | 33.396 | 67.758 | 0.492 |
| Average |  | 33.397 (0.001) | 67.802 (± 0.044) | 0.492 (0.0003) |
| Conf. Interval (95%) |  | 33.39 - 33.40 | 67.72 - 67.89 | 0.49 – 0.49 |
|  |  |  |  |  |
| Group 3 |  |  |  |  |
| scabbardfish-423 | 423* | 26.796 | 75.384 | 0.355 |
| frog-423 | 423* | 28.898 | 65.784 | 0.439 |
| chicken-413 | 415* | 30.572 | 69.654 | 0.439 |
| human-414 | 414* | 31.422 | 60.574 | 0.519 |
| bovine-438 | 438* | 31.918 | 62.522 | 0.510 |
| squirrel-440 | 440* | 31.579 | 60.055 | 0.526 |
| elephant-419 | 419* | 31.153 | 70.007 | 0.445 |
| wallaby-420 | 420* | 32.086 | 61.407 | 0.522 |
| Average |  | 30.553 (± 0.644) | 65.673 (± 1.958) | 0.469 (± 0.021) |
| Conf. interval (95%) |  | 29.29 - 31.82 | 61.84 - 69.51 | 0.43 - 0.51 |

*For the data source, see Materials and methods. The most closely located amino acids at site 91 were considered in place of 86. Scabbardfish-423, AncVertebrate-361 and AncEuteleost-360 all have V91.

AncBird*-393 is identical to AncSauropsid-360 with F49V/F86S/L116V/S118A.
